# CCL20 secreted by KRT15^high^ tumor Cells promotes tertiary lymphoid structure formation and enhances anti-PD-1 therapy response in HPV^+^HNSCC

**DOI:** 10.1038/s41419-025-08359-5

**Published:** 2025-12-29

**Authors:** Siwei Zhang, Huan Liu, Xiaoxing Li, Yourong Jiang, Lu Tang, Tianyang Liu, Rui Li, Zengchen Liu, Minghui Wei, Jingchun Sun, Zhuledesi Hahan, Heng Ma, Lanlan Wei

**Affiliations:** 1 National Clinical Research Center for Infectious Diseases, The Third People’s Hospital of Shenzhen; The Second Hospital Affiliated to Southern University of Science and Technology, Shenzhen, Guangdong 518000 China; 2 The First Affiliated Hospital of Harbin Medical University, Harbin Medical University, School of Stomatology, Harbin, Heilongjiang 150001 China; 3 Department of Pathology, The Third People’s Hospital of Shenzhen, The Second Hospital Affiliated to Southern University of Science and Technology, Shenzhen, Guangdong 518000 China; 4 Department of Stomatology, Jining Medical University, Jining, Shandong 272067 China; 5 Department of Stomatology, Hubei Polytechnic Institute, Xiaogan, Hubei 432000 China; 6 Department of neurosurgery, The Third People’s Hospital of Shenzhen; The Second Hospital Affiliated to Southern University of Science and Technology, Shenzhen, Guangdong 518000 China; 7 Department of Head and Neck Surgery, National Cancer Center/National Clinical Research Center for Cancer/Cancer Hospital & Shenzhen Hospital, Chinese Academy of Medical Sciences and Peking Union Medical College, Shenzhen, 518116 China

**Keywords:** Cancer microenvironment, Immunotherapy, Head and neck cancer

## Abstract

Tertiary lymphoid structures (TLS) are associated with an improved response to Immune checkpoint therapy (ICT) in head and neck squamous cell carcinoma (HNSCC). Human papillomavirus (HPV) infection constitutes a high-risk factor for HNSCC carcinogenesis. However, its role in TLS formation has yet to be elucidated. Herein, immunohistochemical (IHC) analysis from 59 HNSCC patients revealed a higher prevalence of mature TLS in HPV-positive (HPV^+^) HNSCC compared to HPV-negative (HPV^-^) cases. Furthermore, integrated analysis of single-cell RNA sequencing, spatial transcriptomics, and RNA-seq data indicated that TLS-positive tumors were characterized by an expanded population of KRT15^high^ tumor cells in HNSCC. IHC and cytological experiments confirmed upregulation of KRT15 in HPV^+^HNSCC tumor cells, which also showed high expression of cancer stem cell marker genes. These KRT15^high^ stem-like tumor cells specifically secreted CCL20, which was related to the infiltration of TLS-associated immune cells in HPV^+^HNSCC. Murine models confirmed that CCL20 treatment promoted TLS formation and enhanced the efficacy of anti-PD-1 therapy. Multiplex immunofluorescence showed that TLS provided specialized microenvironments that supported the proliferation of CD39^+^PD-1^+^CD8^+^T cells. Collectively, our findings proposed that CCL20 secreted by HPV-infected KRT15^high^ tumor cells promoted TLS formation, thereby enhancing anti-PD-1 therapy responses in HPV^+^HNSCC. This study provides mechanistic insights into HPV-mediated TLS development and supports precision immunotherapeutic strategies for HNSCC.

## Introduction

Head and neck squamous cell carcinoma (HNSCC) is the sixth most prevalent cancer worldwide [1]. Human papillomavirus (HPV) is an important risk factor for HNSCC [1, 2]. Immune checkpoint therapy (ICT) is the first-line therapy for recurrent or metastatic HNSCC [3]. Although ICT is an effective treatment, most patients do not achieve a clinical response to ICT. Recent clinical studies have found that HPV^+^HNSCC usually demonstrated a more favorable prognosis and improved response to ICT compared to HPV^-^HNSCC patients [4–7]. Nevertheless, the mechanisms and causative factors for this phenomenon have not yet been elucidated in current research.

Mature tertiary lymphoid structures (TLS) have been identified as a critical prognostic factor associated with superior outcomes in immunotherapy [8]. TLS are ectopic lymphoid aggregates in the tumor microenvironment (TME), which could facilitate antigen presentation, promote the recruitment of effector immune cells, and support the production of tumor-specific antibodies. Follicular dendritic cells (FDCs) represent a critical hallmark of TLS maturation, whereas immature TLS typically show focal accumulations of T and B cells. Mature TLS features organized aggregates of T cells, B cells, and follicular dendritic cells, while highly mature TLS are characterized by the formation of distinct functional zones within these aggregates, which are pathologically identified as lymphoid follicles [8–11]. Recent studies have demonstrated the presence of mature TLS in HNSCC [4, 12], which have been confirmed to promote patient prognosis and enhance response to immunotherapy [4, 7]. However, the potential impact of HPV infection on TLS development has not yet been investigated.

The development and maturation of TLS rely heavily on the accumulation of chemokines and inflammatory cytokines. These signaling molecules, produced by stromal cells and lymphocytes, attract lymphoid tissue inducer (LTi) cells to inflamed tissues, where they promote TLS formation [13, 14]. For instance, PD-1^+^CXCR5^-^CD4^+^T cell subset drives B cells into TLS of nasopharyngeal carcinoma through CXCL13 [15]. CCL19-producing fibroblasts promote TLS formation in colorectal cancer liver metastasis [16]. Although numerous studies have reported that chemokines secreted by immune cells and stromal cells promoted TLS formation, the potential contribution of tumor cells to this process has received little attention. Currently, the characteristics of tumor cells in TLS-positive (TLS^+^) HNSCC are still unknown. It remains unreported whether HPV-infected tumor cells could influence the TLS formation and consequently modulate the efficacy of ICT in HNSCC.

In this study, we integrated single-cell RNA sequencing(scRNA-seq), spatial transcriptomics (ST), and bulk RNA-seq data from HNSCC with in vitro experiments and murine models to investigate the relationship between HPV-infected tumor cells and TLS formation in HPV^+^HNSCC and the potential mechanisms. These findings provide novel biomarkers for predicting immunotherapy response and potential therapeutic targets in HPV^+^HNSCC.

## Methods

### Patient characteristics

The inclusion criteria for HNSCC patients were as follows: individuals aged 18 years or older who voluntarily agreed to participate in the project and were diagnosed by their attending physician with HNSCC requiring surgical resection. Prior to the surgery, the patient was required to sign an informed consent form. Clinical samples were collected at the National Cancer Center/National Clinical Research Center for Cancer/Cancer Hospital & Shenzhen Hospital, including 25 HPV^+^ and 34 HPV^-^HNSCC samples. Human tissue samples were consecutively enrolled from our clinical tissue biobank without specific selection. All clinical samples of participants were used in the study, i.e., there was no attrition. Ethical approval for the study was obtained from the Ethics Committee of the Third People’s Hospital of Shenzhen [No. 2025-016]. Participant credentials were fully protected.

### Cell lines

Human HNSCC cell line Cal27 and SCC47 cell lines were kindly provided by Dr. Songbin Fu (Harbin Medical University, Harbin, China) and Dr. Henning Willers (Harvard University, Boston, USA). The human HNSCC cell lines were cultured in DMEM supplemented with 10% fetal bovine serum (FBS) and 1% penicillin-streptomycin (P/S). Mouse HNSCC cell line MOC1 cell lines were obtained from FuHeng Biology company (China) and were cultured in IMDM:F12(2:1) supplemented with 10% FBS, 2.5 mg/ml insulin, 20 μg Hydrocortisone, 2.5 μg EGF, and 1%P/S. All cell lines were maintained at 37 °C in a humidified incubator with 5% CO₂. Cell lines were confirmed via short tandem repeat (STR) analysis and tested negative for mycoplasma contamination.

### Mice

Specific-pathogen-free female, 6–8 weeks old, C57BL/6 mice were obtained from GemPharmatech Company (China). All mice were housed under specific pathogen-free (SPF) conditions and were fed with a standard chow diet at the Animal Center in the Third People’s Hospital in Shenzhen. All experimental procedures were approved by the Institutional Animal Care and Use Committee (IACUC) of the Third People’s Hospital of Shenzhen and received ethical approval for the animal research from the Ethics Committee in the Third People’s Hospital of Shenzhen [2025-017-01].

### Animal experiments

MOC1 cells (5 × 10^6^) were subcutaneously injected into the right flank of C57BL/6 mice. The sample size was determined by a power analysis based on pilot data. Blinding of the investigators was not possible due to the obvious visible differences between the treatment and control groups. Tumor volume (mm^3^) was measured with calipers every second day and calculated with the formula (length × width^2^)/2. Prior to the experiment, all mice were consecutively numbered and then initially divided into the CCL20 treatment group and the control group based on odd and even numbers. Recombinant murine CCL20 protein (rmCCL20, HY-P7263, MCE) was administered peritumorally at a dose of 5 μg per mouse starting on the day of tumor inoculation and then every other day until the end of the experiment. On day 10 after tumor inoculation, 3–4 mice from each group were sacrificed for TLS formation detection. To minimize the impact of intra-group variability in tumor volume on the assessment of PD-1 treatment efficacy,mice exclusion was performed prior to PD-1 treatment initiation. We calculated the interquartile range (IQR) for each group, and mice with tumor volumes significantly below (Q1 − 1.5 × IQR) or above (Q3 + 1.5 × IQR) were excluded. The remaining mice were then re-randomized into PD-1 or IgG treatment groups based on the remainder obtained when dividing their assigned serial numbers by 3. Mice were injected intraperitoneally with InvivoMAb rat IgG2a isotype control (100 µg, BE0089; BioXCell) or InvivoMAb anti-mouse PD-1 (200 µg, BE0146; BioXCell). In the combination therapy group, mice were treated with an anti-murine PD-1 antibody with readministration every 3 days for a total of four doses.

### Immunohistochemistry and multiple immunofluorescence

IHC staining was performed as described previously [17]. After deparaffinization and rehydration, the sections were covered with Tris-EDTA (pH 9.0) buffer for antigen retrieval in the microwave at medium power for 10 minutes. Sections were incubated with primary antibodies at 37 °C for 1.5 h and with goat anti-mouse or anti-rabbit secondary antibodies for 1.5 h. All antibodies used in this study, along with their corresponding dilution ratios, are listed in Supplementary Table 1. CD4 and CD19 staining were used to detect the presence of TLS, while CD21 staining served as a marker for mature TLS.

Multiple immunohistochemistry (mIHC) was performed with Opal Polaris 7 Color IHC Detection Kits (Akoya Biosciences, America). After secondary antibody incubation, the staining signal was amplified using the fluorescent dye from the kit. Antigen repair was re-performed by sequentially incubating the sections with different antibodies. The procedure was repeated until all the antibodies were incubated, after which the fluorescent signals were amplified with Opal 520, Opal 570, Opal 620, Opal 650, and Opal 690 dyes. After DAPI staining and sealing, the sections were scanned and analyzed using Akoya Mantra imaging equipment.

### HPV RNA-scope assay

The HPV infection state was detected by an RNAscope 2.5 HD Assay (Advanced Cell Diagnostics, America). FFPE sections (5 μm) were deparaffinized in xylene, followed by dehydration in 100% ethanol. Then, the sections were covered with hydrogen peroxide and incubated in an antigen retrieval reagent. After treatment with protease at 40 °C for 30 min, the sections were hybridized with target HPV16 probes in a HybEZ hybridization oven. The sections were incubated in hybridization buffer at 40 °C following the instructions for signal amplification. Chromogenic detection was performed with DAB, followed by counterstaining with hematoxylin. The endogenous housekeeping gene PPIB was used as a positive control, and the bacterial gene dapB was used as a negative control to assess background signals. Positive staining with signals was visualized under a 20× or 40× objective lens with an Olympus BX51 microscope.

### Data and code information

scRNA-seq data for HNSCC were downloaded from the Gene Expression Omnibus database (GEO, https://www.ncbi.nlm.nih.gov/geo/)under accession numbers GSE139324 and GSE164690. The RNA-seq sequencing data and corresponding clinical information for 518 head and neck cancer patients were downloaded from the Cancer Genome Atlas Program (TCGA, https://www.cancer.gov/ccg/research/genome-sequencing/tcga). The spatial transcriptomic sequencing data of four HNSCC patients and all the code provided in this article can be requested from the corresponding author.

### Single-cell sequencing analysis

scRNA-seq data for CD45^+^ lymphocytes in HNSCC, including 18 HPV^−^ and 8 HPV^+^HNSCC patients, were downloaded from the GEO database under GSE139324. In addition, nine HPV^−^ and six HPV^+^HNSCC scRNA-seq data from the GSE164690 dataset were used to perform cell communication analysis and deconvolution analysis. These samples were first processed with Seurat 3.0 in R (version 4.1.2) to remove low-quality cells. Mitochondrial, ribosomal and hemoglobin genes were filtered. Overall, 20,326 genes were detected in a total of 58,756 cells in GSE139324, and 25,517 genes were detected in a total of 73,568 cells in GSE164690. Then, we utilized functions in the Seurat package to normalize and scale the single-cell gene expression data. Principal component analysis (PCA) was subsequently performed, and 28 or 36 significant principal components were identified for GSE139324 or GSE164690. Cell clustering analysis was carried out with the parameter resolution of 1.2. Cell clusters were annotated with marker genes (Supplementary Table 2).

### Spatial transcriptomic assay and data processing

Two HPV^+^ and two HPV^−^HNSCC TLS^+^ Formalin fixed paraffin embedded (FFPE) tissues were chosen after RNA quality assessment. In addition, the clinical information of these four patients is shown in the table (Supplementary Table 3). The capture area (6.5 × 6.5 mm^2^) in each tissue was performed via a spatial transcriptomics assay (10×Genomics) according to instructions. After library construction, 150 PE mode sequencing was carried out by the Illumina NovaSeq 6000 platform. Primary quality control was conducted with a FASTQ file. After obtaining high-quality clean reads, FASTQ files and manually aligned histology images were analyzed with Space Ranger provided by 10×Genomics Space Ranger following instructions. An average of 2427 spots were measured, with the median number of genes per spot ranging from 1079 to 6471. Spatial transcriptomic data were imported into R via Seurat V.4.0.1 for basic analysis. Cell-cell communication analysis across distinct spots in spatial transcriptomic data was performed using CellChat [18].

### RNA-seq data analysis

RNA-seq sequencing data and clinical information of 518 HNSCC patients were downloaded from the TCGA database, including 421 HPV^-^ and 97 HPV^+^ patients. The sequencing data of these patients were clustered through PCA dimensionality reduction with R, and the patients were divided into five groups. Further analysis of the highly expressed characteristic genes in different patient groups was conducted using the FindAllMarkers functions. Prognostic and characteristic gene expression analyses were performed using the Survival R package or the TIMER2.0 (http://timer.cistrome.org/) database [19].

### TLS signature gene analysis

The high-expression genes were characterized in the TLS region of HPV^+^ and HPV^-^HNSCC by the FindAllMarkers function. These genes were subsequently screened according to the following criteria: Log_2_| fold change| ≥1 and *P* < 0.05. A total of 956 highly expressed genes in the TLS region were obtained (Supplementary Table 4). The spatial distribution of these genes was evaluated in the HPV^-^HNSCC-2 and HPV^+^HNSCC-1 samples. Finally, 10 transcriptional genes were identified to characterize mature TLSs in HNSCC, including CD22, PCLAF, TCL1A, CD72, MYBL2, RGS13, DTX1, PAX5, CD52, and RMI2.

### Plasmid and HPV16 oncogene-expressing stable cell line

The plasmids pcDNA3.1-HPV16 E5, pcDNA3.1-HPV16 E6 and pcDNA3.1-HPV16 E7 were procured from Shanghai Kangcheng Biological Company. All plasmid constructs were verified by DNA sequencing. To establish stable cell lines expressing HPV16 E5, E6, or E7, CAL27 cells were infected with the respective lentivirus and subsequently selected using puromycin. The expression of HPV16 E5, E6, and E7 genes in the cell lines was verified by qRT-PCR.

### RNA extraction and reverse transcription PCR

In brief, reverse transcription was carried out using the Primerscript RT Reagent Kit (Takara, RR047A), and real-time qPCR was performed with TB Green Premixture Ex Taq (Takara, RR820A). Gene expression changes were calculated using the 2^−ΔΔCT^ method relative to the negative control. The forward and reverse primers are shown in Supplementary Table 5.

### Statistical analysis

Statistical analyses comparing two groups were carried out using the nonparametric Mann–Whitney test, chi-square test or two-sided Student’s *t*-test with GraphPad Prism 6 software. In the animal experiment, one-way analysis of variance (ANOVA) was employed to assess whether the differences in tumor volume among the various experimental groups were statistically significant. All tests were chosen after verifying their underlying assumptions. The homogeneity of variance test was conducted using SPSS 27.0.1. Following the confirmation that variances were similar, Student’s *t*-test or ANOVA was performed. Error bars represent mean ± standard deviation (SD) in our results. The SD directly reflects the variability of data points within each experimental group. Each experiment was repeated three times or more to avoid errors. *P < 0.05; **P < 0.01; ***P < 0.001; ****P < 0.0001 indicate a significant difference.

## Results

### High prevalence of mature TLS correlates with improved prognosis in HPV^+^HNSCC

**Fig. 1:**
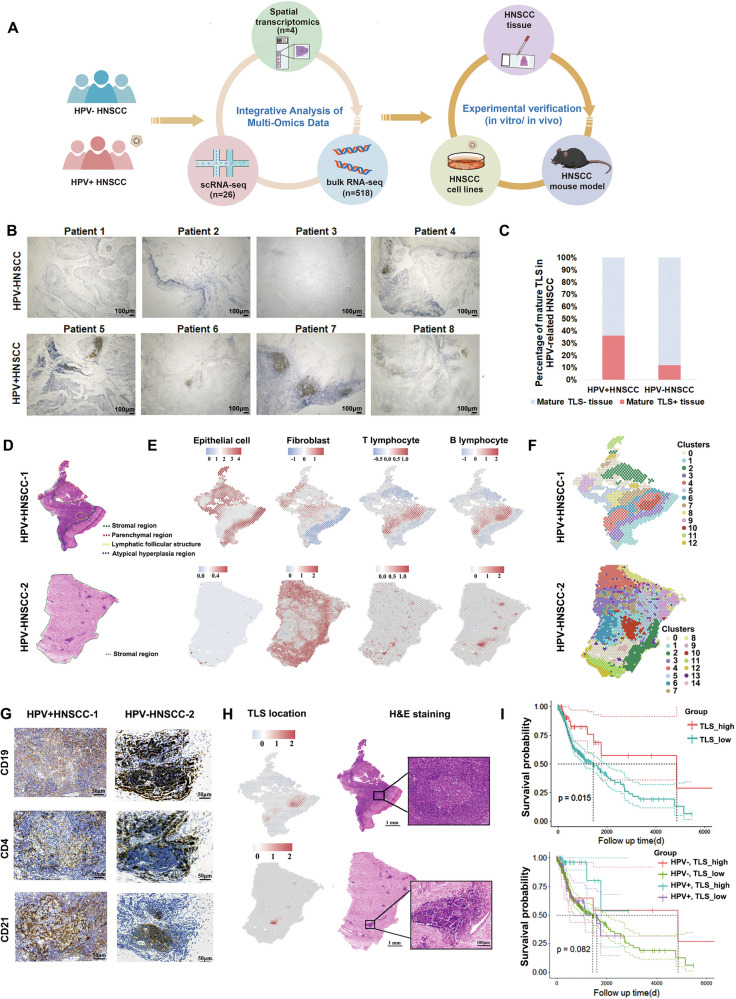
Increased prevalence of mature TLS in HPV^+^HNSCC and positive correlation with prognosis. **A** Schematic diagram of the workflow in this study. **B** Representative IHC images of CD21 (dendritic cell) in HNSCC. **C** Prevalence of mature TLS^+^ cases among HPV^+^ and HPV^−^HNSCC. **D** Histological region classification of HNSCC samples for spatial transcriptome (ST) sequencing. **E** ST analysis of cellular distribution in HNSCC samples. **F** Unsupervised clustering analysis of ST data. **G** IHC staining for mature TLS-associated cellular components. CD4 (T cells), CD19 (B cells), CD21 (dendritic cells). **H** The spatial location of TLS in HNSCC predicted by our transcriptional signature geneset (left). H&E staining of the structural organization in TLS (right). **I** Survival analysis of HPV^+^ and HPV^-^HNSCC patients based on mature TLS prediction score.

To examine the presence and distribution of mature TLS in HPV^+^ and HPV^-^HNSCC, we first collected 25 HPV^+^ and 34 HPV^-^HNSCC tissues for detection (Fig. 1A). HPV infection status was determined by IHC and HPV RNA in situ hybridization (Supplementary Fig. 1A). Then, the presence of follicular dendritic cell networks was confirmed in these tissues, which was the hallmark of mature TLS [8]. The results revealed that HPV^+^HNSCC patients had a higher prevalence of mature TLS than HPV^-^ patients (Fig. 1B, C). To investigate the role of tumor cells in the formation of TLS, two HPV^+^ and two HPV^−^HNSCC tissues were analyzed using 10× Genomics Visium spatial transcriptomics (ST). Tissue sections stained with H&E staining were evaluated by professional pathologists and categorized into distinct pathological regions (Fig. 1D and Supplementary Fig. 1B). The presence of lymphoid follicle structures represents a hallmark pathological feature of mature TLS. Spatial transcriptomic analysis revealed that the distribution of stroma and immune cells within the tumor microenvironment aligned with the distinct pathological regions (Fig. 1E and Supplementary Fig. 1C).

Next, unsupervised clustering of spatial transcriptomic data from four samples were performed (Fig. 1F and Supplementary Fig. 1D). TLS regions were confirmed through IHC and H&E staining evaluation, including cluster 10 in HPV^+^HNSCC-1 and cluster 14 in HPV^−^HNSCC-2 (Fig. 1G, Supplementary Fig. 1E, and Supplementary Table 4). To analyze the characteristic genes of mature TLS in HNSCC, differential gene expression analysis and spatial correlation analysis of TLS were conducted. As a result, ten transcriptional signature genes associated with mature TLS in HNSCC were identified (see Methods). Our defined geneset provided more accurate prediction of mature TLS regions than unsupervised clustering, and the predicted region were consistent with histological results (Fig. 1H and Supplementary Fig. 1F). Additionally, this geneset also effectively predicted TLS areas in publicly available HNSCC spatial transcriptomic datasets (GSE220978) (Supplementary Fig. 1G).

To investigate the correlation between mature TLS and clinical outcomes, our defined TLS geneset was applied to estimate the scores of mature TLS presence in 518 HNSCC patients from the TCGA database. The results revealed that patients with higher mature TLS presence scores had a significantly better prognosis. However, in TLS^+^HNSCC, HPV^+^HNSCC patients still had better prognosis than HPV^-^HNSCC (Fig. 1I). Taken together, these results suggested that TLS formation was more frequently observed in HPV^+^HNSCC patients, which were significantly associated with improved clinical outcomes.

### HPV-regulated KRT15^high^ tumor cells characterize TLS^+^HNSCC

**Fig. 2:**
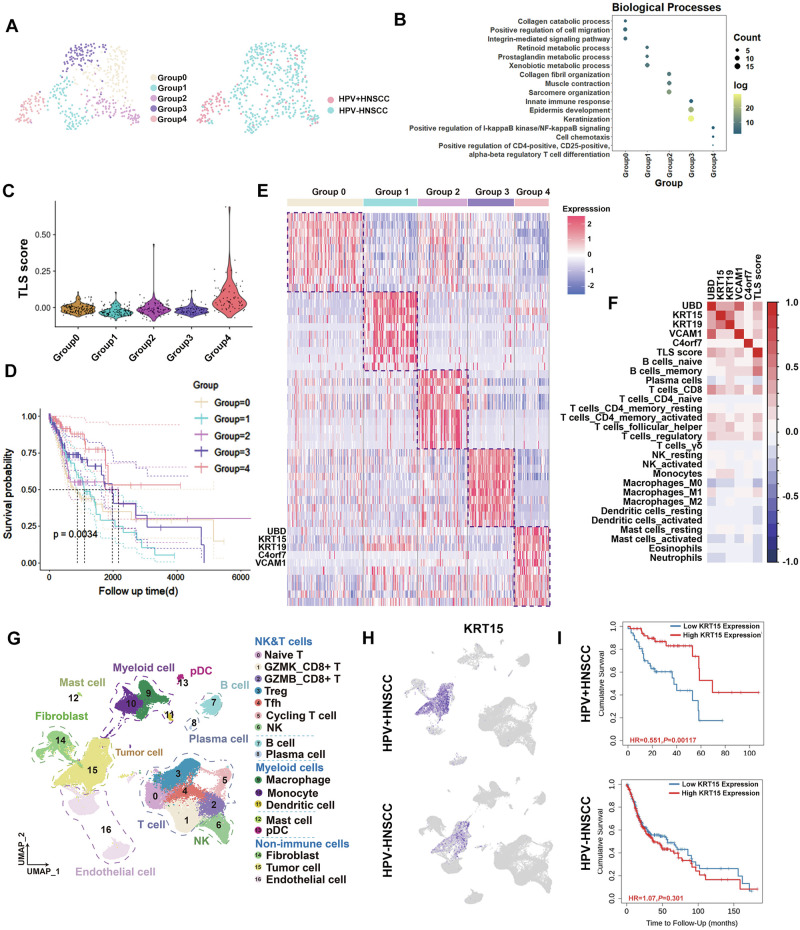
Transcriptomic features of tumor cells in TLS^+^HNSCC patients. **A** Clustering analysis of HNSCC, dividing them into five groups (left), and HPV infection status of the patients (right). **B** Functional enrichment analysis of characteristic genes in HNSCC across different groups. **C** Mature TLS prediction score across different groups. **D** Survival analysis of patients from different groups. **E** Heatmap analysis of highly expressed characteristic genes in distinct groups. **F** Correlation analysis between the highly expressed genes in Group 4, immune cell infiltration, and mature TLS prediction score. **G** UMAP of the tumor microenvironment landscape in HNSCC. **H** Spatial distribution of KRT15 expression in HPV^+^ and HPV^−^HNSCC microenvironments with scRNA-seq data. **I** Prognostic correlation analysis of KRT15 in HPV^+^ and HPV^−^HNSCC. *P < 0.05; **P < 0.01; ****P < 0.0001.

To further explore the characteristics of TLS^+^patients, RNA-seq data from 518 HNSCC patients in the TCGA database were analyzed and stratified into five distinct groups. Notably, the majority of patients in Group 4 were HPV^+^HNSCC patients (Fig. 2A) and characterized by high levels of CD8^+^T cells infiltration (Supplementary Fig. 2A). Functional enrichment analysis of highly expressed differentially genes in each group revealed significant activation of NF-κB signaling and cell chemotaxis pathways in Group 4 patients (Fig. 2B). Next, we assessed the presence of mature TLS in these five groups and the results demonstrated a higher probability of mature TLS formation in Group 4 patients (Fig. 2C). Survival analysis also revealed that patients in Group 4 had a better prognosis (Fig. 2D). Overall, these observations identified that patients in Group 4 were candidates for mature TLS formation.

Therefore, we focused subsequent analysis on Group 4 and discovered the significant overexpression of UBD, KRT15, KRT19, C4orf7 and VCAM1 in Group 4 patients (Fig. 2E). Correlation analysis revealed that the expression of UBD, KRT15, KRT19 and VCAM1 were associated with the high infiltration of multiple immune cells, including CD8^+^T cells, and the presence of TLS (Fig. 2F). Additionally, the expression of these five genes were significantly elevated in HPV^+^HNSCC compared to HPV^-^HNSCC patients (Supplementary Fig. 2B).

To further investigate the characteristics of tumor cells in TLS^+^ patients, we examined the distribution of UBD, KRT15, KRT19 and VCAM1 in different cells within the tumor microenvironment. Firstly, scRNA-seq data were obtained from 9 HPV^-^ and 6 HPV^+^HNSCC patients in the GEO database (GSE164690). After quality control, a total of 73,568 high-quality cells were obtained and subsequently clustered into 17 subtypes (Fig. 2G). The infiltration ratios of B cells and CD8^+^T cells were higher in HPV^+^HNSCC (Supplementary Fig. 2C). The signature genes were shown in Supplementary Fig. 2D and Supplementary Table 2. Then, scRNA-seq analysis revealed that KRT15 and KRT19 were exclusively distributed in tumor cells, especially in HPV^+^HNSCC (Fig. 2H and Supplementary Fig. 2E). KRT15 and KRT19 are keratin proteins that play essential roles in epithelial cell differentiation and tumorigenesis [20, 21]. We further assessed the correlation of KRT15 and KRT19 expression with clinical outcomes in both HPV^+^ and HPV^-^HNSCC patients. The results showed that high KRT15 expression was positively associated with better prognosis in HPV^+^HNSCC rather than HPV^-^HNSCC. However, no significant association was observed between KRT19 expression levels and clinical outcomes in HNSCC patients (Fig. 2I and Supplementary Fig. 2F). In summary, these results indicated that tumor cells with high KRT15 expression improve the prognosis of HPV^+^HNSCC patients and were associated with the presence of mature TLS.

**Fig. 3:**
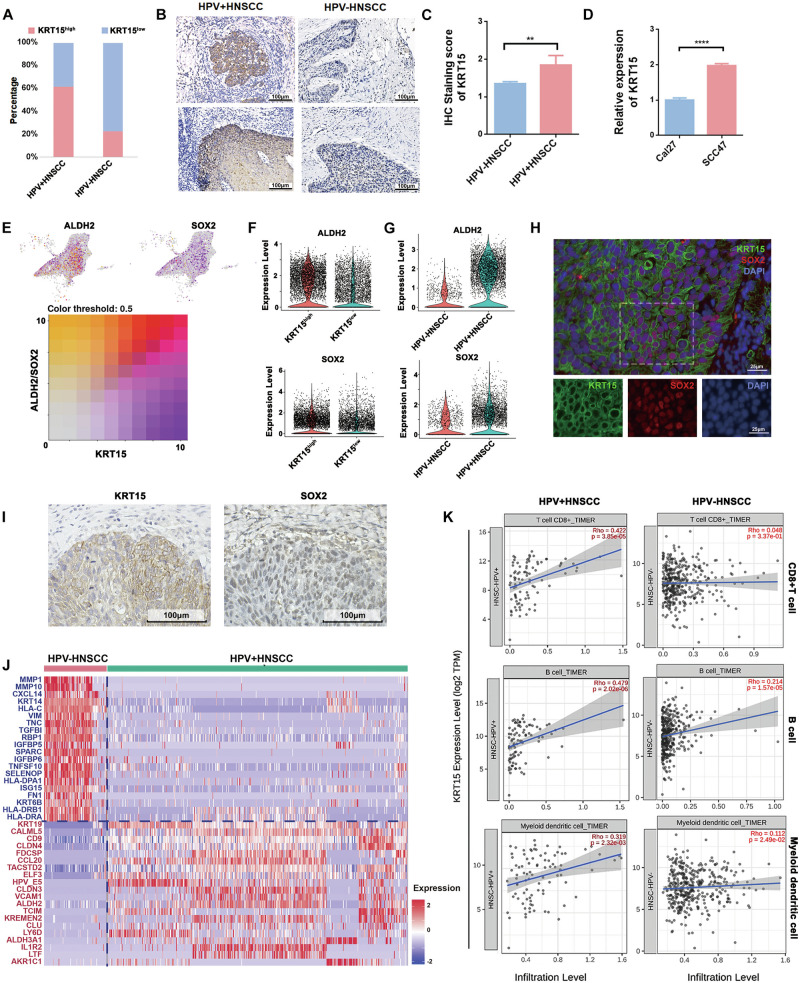
Characterization and biological functions of KRT15^high^ tumor cells in HNSCC. **A** Proportion of KRT15^high^ tumor cells in HPV^+^ and HPV^-^HNSCC. **B** Representative IHC images of KRT15 detection in HPV^+^ and HPV^-^HNSCC. **C** Statistical analysis of IHC detection results for KRT15. **D** Detection of KRT15 in HPV^+^ and HPV^−^HNSCC cell lines. **E** Co-expression of KRT15 and stem-cell markers (ALDH2 and SOX2) identified by scRNA-seq. **F** Expression of stem-cell marker in KRT15^high^ vs KRT15^low^ cells with scRNA-seq data. **G** Expression of stem cell markers in KRT15^high^ cells from HPV^+^ and HPV^-^HNSCC. **H** Representative immunofluorescence (IF) staining showing colocalization of KRT15 and SOX2 in HPV^+^HNSCC. **I** Representative IHC images of KRT15 and SOX2 in HPV^+^HNSCC. **J** Heatmap of transcriptional differences in KRT15^high^ tumor cells between HPV^+^ and HPV^-^HNSCC. **K** Correlation analysis between KRT15 expression and immune cell infiltration in HNSCC. **P < 0.01; ****P < 0.0001.

We next divided the tumor cells into KRT15^high^ and KRT15^low^ expression groups and found distinct gene expression profiles between these two groups (Supplementary Fig. 3A). Furthermore, both KRT19 and UBD were significantly upregulated in the KRT15^high^ group, with particularly high expression in KRT15^high^ cells of HPV^+^HNSCC (Supplementary Fig. 3B). ScRNA-seq analysis demonstrated that the percentage of KRT15^high^ tumor cells was significantly higher in HPV^+^HNSCC than in HPV^-^HNSCC (Fig. 3A). KRT15 expression were further detected by IHC in HNSCC tissues and the results showed the expression of KRT15 was upregulated in HPV^+^HNSCC (Fig. 3B, C). In situ analysis also confirmed the presence of HPV16 RNA within KRT15^high^ tumor cells in HPV^+^HNSCC (Supplementary Fig. 3C). Similar results were observed in both HPV^+^ and HPV^-^ cell lines (Fig. 3D). The HPV E5, E6, and E7 are common oncogenes in tumor development [2].To identify the key viral gene affecting KRT15 expression, we separately transfected HPV^-^HNSCC cell line CAL27 with HPV16 E5, HPV16 E6, and HPV16 E7 plasmids (Supplementary Fig. 3D). The results showed that the expression of KRT15 was increased in the Cal27-E6 cell line (Supplementary Fig. 3E). Collectively, these results indicated that HPV infection could promote KRT15 expression in tumor cells.

Epithelial tissue is organized into multi-layers, including the basal cell layer, spinous layer, granular layer, and keratinized layer, with tumor stem cells predominantly located in the basal layer [22]. To investigate whether KRT15^high^ cells showed tumor stem cell properties, we assessed the expression of cancer stem cell markers in KRT15^high^ tumor cells, including ALDH2, SOX2, CD44 and DKK3. Gene expression analysis revealed that the expression of ALDH2 and SOX2 were upregulated in KRT15^high^ cells (Fig. 3E, F), while the expression of CD44 was elevated in KRT15^low^ cells (Supplementary Fig. 3F). Furthermore, scRNA-seq analysis revealed that the expression of ALDH2 and SOX2 were higher in KRT15^high^ tumor cells of HPV^+^HNSCC (Fig. 3G and Supplementary Fig. 3G). Co-expression of SOX2 was identified in KRT15^high^ cells within HPV^+^HNSCC by immunofluorescence (IF) and IHC (Fig. 3H, I). These results suggested that HPV modulated cancer stem cell characteristics and KRT15^high^ tumor cell showed stem cell-like properties in HPV^+^HNSCC.

In addition, copy number variation (CNV) analysis confirmed that KRT15^high^ tumor cells in HPV^+^ and HPV^-^HNSCC had distinct genetic backgrounds (Supplementary Fig. 3H). In HPV^+^HNSCC, KRT15^high^ tumor cells showed increased expression of genes such as CLDN4, FDCSP, CCL20, and IL1R2 (Fig. 3J). Correlation analysis revealed a positive association between KRT15 expression levels and the cellular components of TLS (CD8⁺T cells, dendritic cells, and B cells) in HPV^+^HNSCC. However, no such correlation was observed in HPV^-^HNSCC (Fig. 3K). Together, these results indicated that KRT15^high^ tumor cells served as a key feature of TLS^+^HNSCC patients and HPV infection promoted the expression of KRT15. These observations suggested that HPV facilitated TLS formation through KRT15^high^ tumor cells.

### KRT15^high^ tumor cell-derived CCL20 relates to TLS formation in HPV^+^HNSCC

**Fig. 4:**
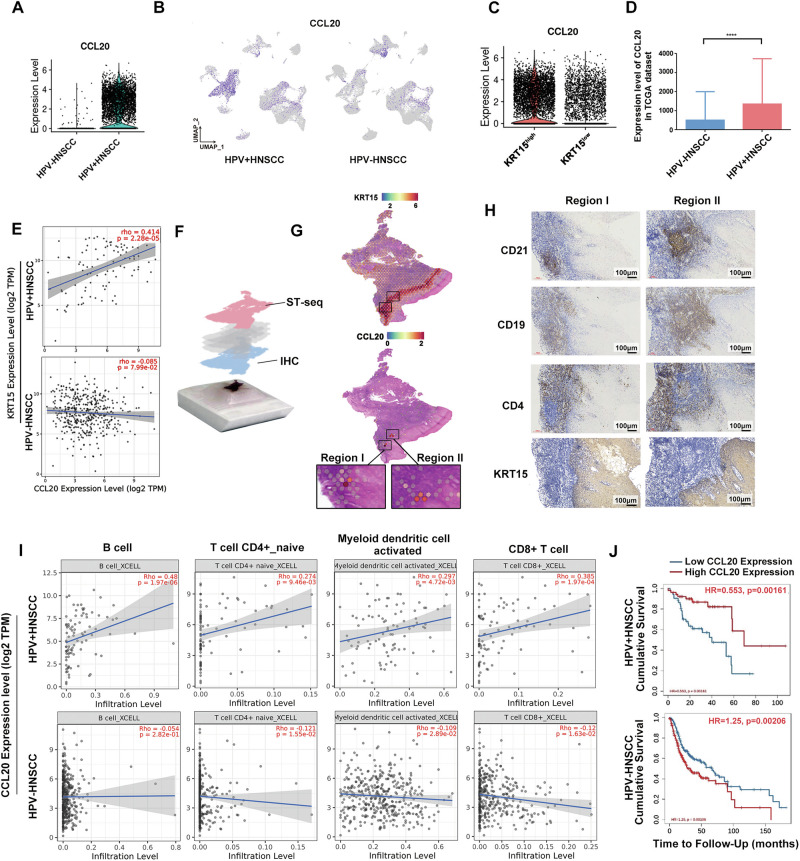
CCL20 secreted by KRT15^high^ tumor cells related to TLS formation in HPV^+^HNSCC. **A** Expression of CCL20 in KRT15^high^ tumor cells from HPV^+^ and HPV^-^HNSCC. **B** Spatial distribution of CCL20 expression in HPV^+^ and HPV^-^HNSCC with scRNA-seq data. **C** Expression of CCL20 in KRT15^high^ and KRT15^low^ epithelial cells in HNSCC. **D** Expression of CCL20 in HPV^+^ and HPV^−^HNSCC with RNA-seq profile from TCGA dataset. **E** Correlation between KRT15 expression and CCL20 in HNSCC. **F** Schematic diagram of spatial transcriptome sequencing (ST-seq) and IHC detection. **G** Expression region of KRT15 and CCL20 in HPV^+^HNSCC based on ST data. **H** IHC detection of mature TLS-associated cellular components and KRT15 expression. **I** Correlation between CCL20 expression and TLS-associated immune cell infiltration in HNSCC. **J** Correlation between CCL20 expression and prognosis in HPV^+^ and HPV^−^HNSCC. ****P < 0.0001.

The CCL20-CCR6 axis serves as a key regulator of immunological responses [23]. During the analysis, we found that CCL20 secretion by KRT15^high^ tumor cells was significantly elevated in HPV^+^HNSCC, while in HPV^-^HNSCC, CCL20 was mainly secreted by myeloid cells (Fig. 4A–C). RNA-seq analysis of HNSCC identified significantly elevated CCL20 expression in HPV^+^HNSCC relative to HPV^-^HNSCC (Fig. 4D). Significant positive correlation was detected between KRT15 and CCL20 expression in HPV^+^HNSCC, whereas no such trend was observed in HPV^-^HNSCC (Fig. 4E). These findings suggested that in HPV^+^HNSCC, CCL20 was specifically secreted by KRT15^high^ tumor cells and contributed to improved patient prognosis.

To further investigate the correlation between CCL20 and TLS, we analyzed ST data and found that CCL20 was highly expressed in KRT15^high^tumor cells surrounding TLS in HPV^+^HNSCC (Fig. 4F, G). Although typical lymphoid follicle structures were not apparent in H&E staining sections of Region I and Region II (Fig. 1D), previous ST analysis revealed elevated expression of mature TLS signature genes (Fig. 1H). Subsequent IHC analysis confirmed the presence of mature TLS structures in Region I and Region II (Fig. 4H). These results suggested a potential association between CCL20 and TLS formation.

To determine the principal cell populations recruited by CCL20, we collected 18 HPV^-^ and 8 HPV^+^HNSCC scRNA-seq data from GSE139324 and clustered them into 7 distinct immune cell populations (Supplementary Fig. 4A, B). CCR6 expression was found to be enriched in T lymphocytes, B lymphocytes, and myeloid cell populations (Supplementary Fig. 4C). Transcriptomic analysis additionally verified the correlation between elevated CCL20 levels and enhanced infiltration of various TLS-associated immune cells in HPV^+^HNSCC (Fig. 4I). High expression of CCL20 was positively correlated with patient prognosis in HPV^+^HNSCC (Fig. 4J).

Furthermore, we similarly employed PCR to assess CCL20 expression in HPV oncogene-transfected cell lines. The results revealed the expression of CCL20 was elevated in CAL27-E6 cells and CAL27-E7 cells, while was reduced in CAL27-E5 cells, indicating that differential HPV oncogene transcription modulated CCL20 expression in tumor cells (Supplementary Fig. 4D). Overall, these findings indicated that in HPV^+^HNSCC, KRT15^high^ tumor cells recruited TLS-associated immune cell through CCL20 secretion.

### CCL20 promotes TLS formation and enhances the efficacy of anti-PD-1 therapy in HNSCC mouse model

**Fig. 5:**
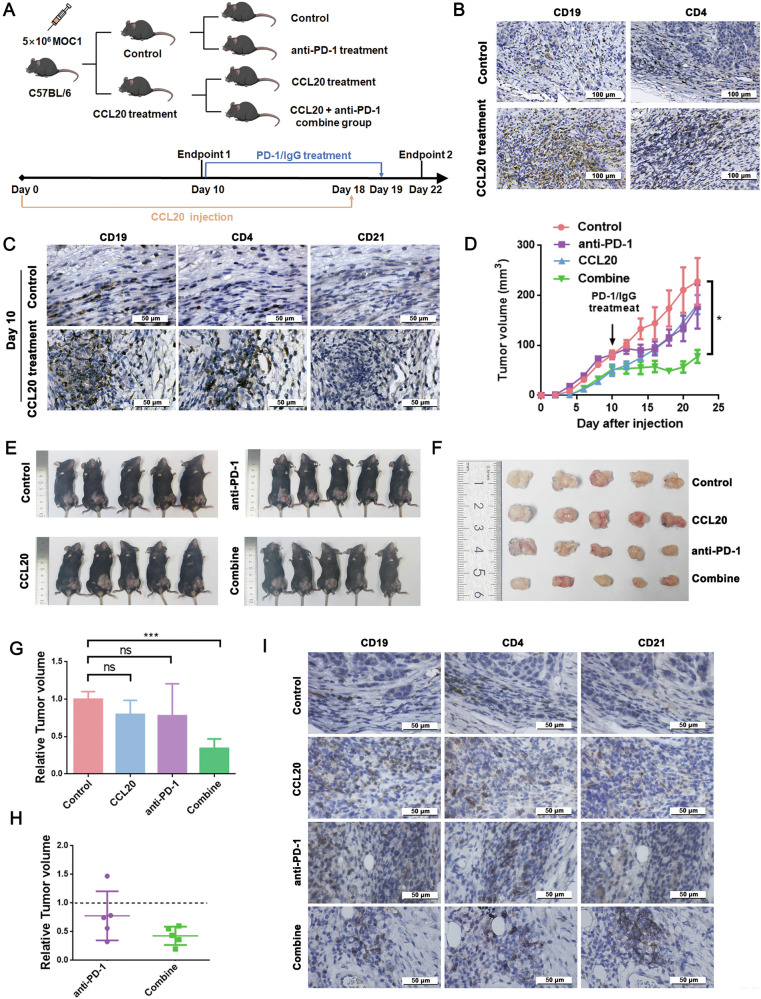
CCL20 treatment promotes TLS formation and enhances anti-PD-1 therapy efficacy in murine models. **A** Schematic diagram of the animal experimental design. **B** Representative IHC images showing the infiltration of CD19^+^B cells and CD4^+^T cells in control and CCL20 treatment groups at day 10. **C** IHC detection of mature TLS-associated cellular components at day 10. **D** Tumor growth curves under different treatments in murine models. *n* = 5 per group. **E** Gross images of mice at day 22. **F** Tumor size assessment across experimental groups at day 22. **G** Tumor volume ratios of CCL20, anti-PD-1, and combination therapy groups relative to the control group. **H** Relative tumor volume ratios between control vs anti-PD-1 groups, and CCL20-treated vs combination therapy groups. **I** IHC detection of mature TLS-associated cellular components at day 22. CD19 (B cells), CD4 (T cells), CD21 (dendritic cells). *P < 0.05, ***P < 0.001.

To explored the effect of CCL20 on TLS formation in vivo, we established HNSCC mouse models using the MOC1 cell line (Fig. 5A). Treatment of CCL20 significantly retarded tumor growth during the early experimental phase (Supplementary Fig. 5A, B). IHC analysis on day 10 of treatment revealed that the infiltration of CD4^+^T cells and CD19^+^B cells were significantly higher in the CCL20 treatment group (*n* = 4) compared to the control group (*n* = 3) (Fig. 5B and Supplementary Fig. 5C). We observed colocalization between CD4^+^T cells and CD19^+^B cell clusters in CCL20 treatment group (Fig. 5C), which have been regarded as markers of TLS formation [8]. CD4⁺ T cells and CD19^+^B cells were showed scattered distribution in the control group (Fig. 5C).

The presence of TLS improves response to immunotherapy in various tumor [24, 25]. We initiated the anti-PD-1 treatment on day 10 after tumor cell injection. Treatment of HNSCC mice with the combination of CCL20 and anti-PD-1 significantly inhibited tumor growth (Fig. 5D-F). Surprisingly, no significant difference in tumor volume was observed between the control group and either the anti-PD-1 or CCL20 treatment group (Fig. 5G). To evaluate the differential response of anti-PD-1 therapy in CCL20-pretreated versus untreated groups, we analyzed the relative tumor volume ratios between control/anti-PD-1 and CCL20/combine treatment groups. The results showed that HNSCC mice receiving CCL20 pretreatment showed a higher response to subsequent anti-PD-1 therapy (Fig. 5H). Comparative analysis of H&E staining across the four groups demonstrated markedly enhanced immune cell infiltration in the combine therapy group relative to others (Supplementary Fig. 5D). The co-localization of CD21^+^dendritic cells, CD4^+^T cells, and CD19^+^B cells were investigated in the combine therapy group and CCL20 treatment group, indicating the formation of mature TLS (Fig. 5I). Taken together, our experimental results demonstrated that CCL20 promoted the formation of mature TLS in HNSCC mice and enhanced anti-PD-1 therapy efficacy.

### Central roles of TLS on PD-1^+^CD8^+^T cells proliferation in HPV^+^HNSCC

**Fig. 6:**
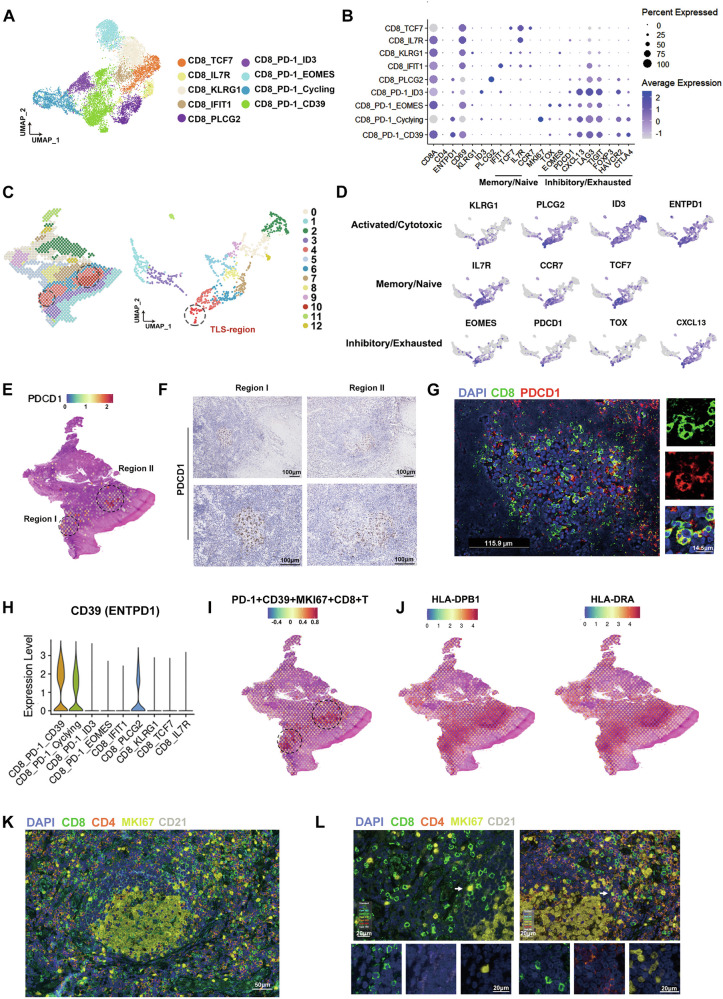
TLS contribute to proliferation of PD-1^+^CD8^+^T cells. **A** Clustering analysis of CD8^+^T cell subsets in HPV^+^ and HPV^−^HNSCC based on scRNA-seq data. **B** Bubble plot of marker genes for T cell subsets. **C** Clustering analysis of HPV^+^HNSCC based on spatial transcriptomics (ST) data. The circled regions indicate the groups containing mature TLS. **D** Expression of T cell subsets signature genes. **E** Spatial distribution of PDCD1 (PD-1) in HPV^+^HNSCC ST data. **F** IHC detection of PD-1 expression in TLS. **G** Immunofluorescence detection of PD-1^+^CD8^+^T cell infiltration in the TLS regions. **H** Violin plot showing the expression of ENTPD1 (CD39) in T cell subsets based on scRNA-seq data. **I** Spatial distribution of PD-1^+^CD39^+^MKI67^+^CD8^+^T cells in HPV^+^HNSCC ST data. **J** Spatial expression of antigen presentation pathway-related genes in HPV^+^HNSCC. **K** Multiplex immunofluorescence visualization of immune cell accumulation in TLS compartments. **L** Immunofluorescence detection of MKI67^+^CD8^+^T cell, CD21^+^dendritic cells and CD4^+^T cells within TLS.

CD8⁺T cells are key antitumor effectors in the tumor microenvironment and the main targets of anti-PD-1 therapy [26]. In vivo experiments revealed that TLS formation suppressed tumor growth and enhanced the efficacy of anti-PD-1 therapy. Based on these findings, we next investigated the potential correlation between TLS and PD-1^+^CD8^+^T cells. We first extracted CD8^+^T cells from 18 HPV^−^ and 8 HPV^+^HNSCC scRNA-seq data, which were then divided into 9 groups (Fig. 6A). Four of these groups consisted of PD-1^+^CD8^+^T cells that highly expressed exhaustion marker genes (LAG3, TIGIT, and CXCL13). Additionally, the high expression of characteristic genes for each subgroup was shown (Fig. 6B). Among them, CD8_TCF7 shared characteristics of naïve T cells (CCR7, IL7R, and TCF7), while CD8_IL7R showed characteristics of tissue-resident central memory T cells (CD69, IL7R). ST data showed that the expression of PD-1 (PDCD1) and CD8 were upregulated in TLS regions (Fig. 6C-E). IHC and IF detection confirmed that PD-1^+^CD8^+^T cells were highly enriched in mature TLS (Fig. 6F,G).

Interestingly, we identified a proliferative subset of PD-1^+^CD8^+^ T cells that expressed high levels of CD39 (ENTPD1) and predominantly localized within mature TLS (Fig. 6H,I). CD39 is recognized as a marker gene for antitumor CD8⁺T cells [27]. Antigen presentation is necessary for the expansion of specific CD8^+^T cells [28]. In TLS of HPV^+^HNSCC, antigen presentation pathway-related genes were upregulated, and cell communication analysis revealed highly active antigen presentation signaling within these structures (Fig. 6J and Supplementary Fig. 6). IF analysis confirmed that MKI67^+^CD8^+^T cells were predominantly localized within TLS regions and showed direct cellular contacts with both dendritic cells and CD4^+^T cells (Fig. 6K, L). These observations suggested that TLS provided specialized microenvironments that supported the proliferation of CD39^+^PD-1^+^CD8^+^T cells.

**Fig. 7:**
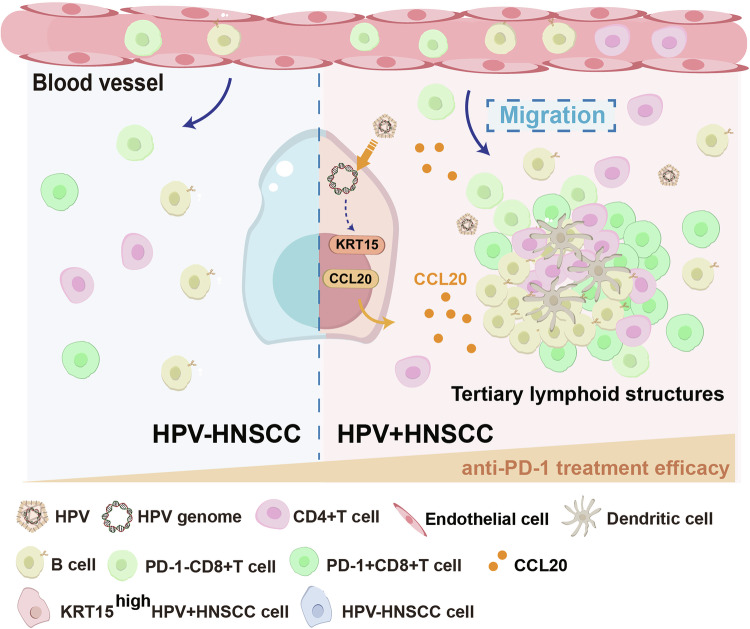
Schematic diagram of study. In HPV^+^HNSCC, CCL20 secreted from KRT15^high^ tumor cells promotes the formation of TLS and enhance anti-PD-1 therapy efficacy. TLS also provide a suitable environment for the proliferation of PD-1^+^CD8^+^T cells.

In conclusion, this study demonstrates that CCL20 secreted by HPV-infected KRT15^high^ stem-like tumor cells promoted the formation of mature TLS in HNSCC and enhanced the efficacy of anti-PD-1 therapy (Fig. 7).

## Discussion

KRT15^high^ tumor cells were located in the basal layer of squamous epithelial cells and highly express tumor stem cell marker genes in our study. Cancer stem cells are defined as self-renewing, multipotent cells capable of giving rise to all differentiated lineages within a tissue [29]. KRT15 is considered a marker gene for long-lived cell populations in esophageal squamous epithelial cells, with these KRT15^high^ expressing cells being able to self-renew, proliferate, and produce differentiated cells [30]. Moreover, in lung epithelium, KRT15^+^ cells show self-renewal and differentiation potential, playing a role in the maintenance, proliferation, and differentiation of airway basal cells [31]. scRNA-seq analysis have revealed that KRT15^+^ cells are actively dividing progenitor cells in lung epithelium [32].

Viral infection serves as a potential factor influencing KRT15 expression. Previous studies have reported Varicella-zoster virus (VZV) infection upregulated the expression of KRT15, which is essential for replication in the skin [33]. In our study, the elevated expression of KRT15 was observed in HPV^+^HNSCC, compared to HPV^-^HNSCC. An HPV16 E6/E7-expressing mouse model revealed that oncogene expression promoted the expansion of KRT15^high^ cells, which was generated by disrupting the balance between stem cell self-renewal and differentiation [34]. Our results demonstrate that HPV16 upregulates KRT15 expression in HNSCC cell lines primarily through the E6. The E6 primarily promotes tumorigenesis by targeting p53 for ubiquitin-mediated degradation and then promote cell proliferation [35]. The p53 proteins are key regulators of stem cell self-renewal in skin tissues [34], which could impact KRT15 expression. It remains to be investigated whether other HPV oncogenic elements, such as the upstream regulatory region (URR) or E1-E4 genes, also impact KRT15 expression. Nevertheless, compared to HPV^-^HNSCC, HPV infection appears to provide new opportunities for antitumor immune responses in HNSCC.

KRT15^high^ tumor cells influences local immune activity in various cancers. In our study, high KRT15 expression correlated with increased infiltration of T cells, B cells, and myeloid dendritic cells, which related to TLS. Previous study showed that high KRT15 expression was significantly associated with IL-27 pathway in breast invasive carcinoma, which enhanced the functions of Th1 and CD8^+^ T cells and promotes the development of follicular helper T cell and B cells [36]. Elevated KRT15 expression correlates with lymphovascular invasion in endometrial cancer [37]. Another research showed that KRT15 epithelial cells were highly enriched in TNF inflammatory factors, and activate the downstream inflammatory functions in cystitis glandularis [38]. TNF-α can induce stromal cells and epithelial cells to express intercellular adhesion factor (ICAM-1), promoting the migration of neutrophils and T cells in the inflammatory environment [39].

TLS have been found in a variety of tumors, and in most cases, their presence are associated with better prognosis in cancer patients. In this study, we demonstrated that HPV-infected KRT15^high^-expressing epithelial cells secreted CCL20, promoting the formation of TLS in HNSCC. CCL20, also known as macrophage inflammatory protein-3α, can be secreted by macrophages, epithelial cells, dendritic cells, and others. The specific receptor for CCL20 is CCR6, which is primarily expressed on immune cells such as Th17, Treg, CD8^+^T cells, and B cells [40, 41]. The level of CCL20 in cervical cancer tissue is significantly higher than in non-tumor and normal control tissues, and it can recruit Th17 cells to accumulate [42]. Th17 cells have been shown to be able to substitute for s lymphoid tissue inducer cells in the initiation of TLS formation [8]. This may explain the formation of TLSs around CCL20^high^ expressing epithelial cells. Additionally, our study found that in HPV^-^HNSCC, CCL20 is mainly secreted by macrophages, and high infiltration of CCL20 is associated with poor prognosis. This could be due to the different immune cell populations recruited by CCL20 from various microenvironments. Studies have shown that CCL20 secreted by tumor-associated macrophages (TAMs) could recruit Treg cells, which might lead to tumor progression and poor prognosis in various cancers [23].

Our study demonstrated that CCL20 treatment enhanced the efficacy of anti-PD-1 therapy in HNSCC by promoting the formation of mature TLS. The clinical impact of TLS on response to immunotherapy has very recently been extended to various cancers, including carcinomas, melanomas, soft tissue sarcomas, and non-small-cell lung cancer [8, 9]. TLS induction has become an important research direction for enhancing therapeutic efficacy. However, current research on TLS applications still faces numerous limitations. Firstly, TLS detection relies on examining tumor samples from specific regions, making it impossible to assess TLS presence in samples that cannot be surgically obtained. Secondly, there is currently no standardized method for TLS quantification. The heterogeneity of TLS across different tumor types poses challenges for its quantification and evaluation. Finally, TLS formation remains dependent on the tumor microenvironment; in pre-established “immune desert” tumors, it is difficult to induce TLS formation and achieve therapeutic benefits.

## Conclusions

In summary, our study revealed that TLS were more frequently observed in HPV^+^HNSCC. Their presence was associated with the upregulation of KRT15 in tumor cells. Moreover, the expression of KRT15 was generally higher in HPV^+^HNSCC, and these KRT15^high^ tumor cells were confirmed to possess stem cell-like characteristics. We further demonstrated that CCL20 secreted by KRT15^high^ tumor cells promoted the formation of mature TLS, which in turn enhances the efficacy of anti-PD-1 therapy in HPV^+^HNSCC.

## Supplementary information

Supplementary Figure legends

Supplementary Table 1-5

Supplementary Figure 1

Supplementary Figure 2

Supplementary Figure 3

Supplementary Figure 4

Supplementary Figure 5

Supplementary Figure 6

## Data Availability

The scRNA-seq data analyzed in this study were obtained from the Gene Expression Omnibus (GEO) at GSE139324 and GSE164690. RNA-seq data were obtained from The Cancer Genome Atlas (TCGA). The ST-seq data generated in this study are available upon request from the corresponding author.
