# Supplementary material for: CCL20 secreted by KRT15high tumor Cells promotes tertiary lymphoid structure formation and enhances anti-PD-1 therapy response in HPV+HNSCC
Source: Cell Death Dis. 2025 Dec 29;17(1):150. doi: 10.1038/s41419-025-08359-5 (PMC12858956; doi:10.1038/s41419-025-08359-5)
Supplement: Supplementary file 1 — Supplementary Figure legends [file 41419_2025_8359_MOESM1_ESM.docx]

**Supplementary Figure 1. Characterization of mature TLS in HPV^+^ and HPV^-^HNSCC.**

(A) IHC and RNA-scope detection of HPV infection status in HNSCC tissues.(B) Histological region classification of HNSCC samples for spatial transcriptome (ST) sequencing.(C) ST analysis of cellular distribution in HNSCC samples.(D) Unsupervised clustering analysis of ST data. (E) IHC staining for mature TLS-associated cellular components. CD4 (T cells), CD19(B cells).CD21(dendritic cells). (F) Prediction of the spatial location of TLS in HNSCC using our transcriptional signature geneset (left). H&E staining of the structural organization in TLS (right). (G) The spatial location of TLS predicted by our transcriptional signature geneset with the public HNSCC spatial transcriptomics dataset.

**Supplementary Figure 2. Transcriptomic features of TLS^+^HNSCC.** (A) Assessment of immune cell infiltration and expression of immune checkpoint genes in patients from different groups. (B) Expression levels of UBD, KRT15,KRT19,C4ORF7 and VCAM1 in HNSCC with RNA-seq profile.(C) ScRNA-seq analysis of immune cell infiltration proportions in HPV^+^ versus HPV^-^ tumor microenvironments.(D) Bubble plot of marker genes for immune cells.(E) Spatial distribution of UBD, KRT19 and VCAM1 expression in HPV^+^ and HPV^-^HNSCC microenvironments with scRNA-seq data.(F) Prognostic correlation analysis of UBD and KRT19 in HPV^+^ and HPV^-^HNSCC.

**Supplementary Figure 3. Characterization of KRT15^high^ tumor cells in HPV^+^ and HPV^-^HNSCC.** (A) Heatmap of differentially expressed genes between KRT15^high^ and KRT15^low^ tumor cells. (B) Expression of UBD and KRT19 in KRT15^high^ versus KRT15^low^ tumor cells in HNSCC(left). Comparative analysis of UBD and KRT19 expression in KRT15^high^ cells from HPV^+^ and HPV^-^ HNSCC(Right). (C) RNA-Scope analysis of HPV16 RNA expression and IHC detection of KRT15 expression in corresponding regions of HPV^+^HNSCC. (D) PCR detection of HPV16 oncogene expression in stably transfected cell lines. (E) PCR detection of KRT15 expression in HPV16 oncogene-stably transfected cell lines. (F) Expression of CD44 and DKK3 in KRT15^high^ versus KRT15^low^ tumor cells in HNSCC. (G) Comparative analysis of CD44 and DKK3 expression in KRT15^high^ cells from HPV^+^ and HPV^-^ HNSCC. (H) CNV analysis of copy number variations in KRT15^high^ and KRT15^low^ epithelial cells in HPV^+^ and HPV^-^HNSCC.

**Supplementary Figure 4.** **Functional and expression analysis of CCL20 in HNSCC.** (A) UMAP analysis of the immune landscape in HNSCC with scRNA-seq data. (B) Bubble plot of marker genes for immune cells. (C) Expression of CCR6 across different immune cell types. (D) PCR detection of CCL20 expression in HPV16 oncogene-stably transfected cell lines.

**Supplementary Figure 5. CCL20 promotes TLS formation in HNSCC murine models.** (A) Tumor growth curves of murine models in control and CCL20 treatment groups. (B) Assessment of tumor volume at day 10. (C) Quantitative IHC comparison of CD4 and CD19 expression between control and CCL20 treatment groups on day 10.(D) Representative H&E staining of individual mice from different treatment groups at day 22.

**Supplementary Figure 6.** **Cell-cell communication analysis between TLS regions and adjacent areas with HPV^+^HNSCC spatial transcriptomics data.**
