# Supplementary material for: CCL20 secreted by KRT15high tumor Cells promotes tertiary lymphoid structure formation and enhances anti-PD-1 therapy response in HPV+HNSCC
Source: Cell Death Dis. 2025 Dec 29;17(1):150. doi: 10.1038/s41419-025-08359-5 (PMC12858956; doi:10.1038/s41419-025-08359-5)
Supplement: Supplementary file 2 — Supplementary Table 1-5 [file 41419_2025_8359_MOESM2_ESM.docx]

**Supplementary Table 1. Information of experiment reagents or resources.**

| **Reagent or Resource** | **Source** | **Identifier** | **Dilution** |
| --- | --- | --- | --- |
| Anti-CD19 antibody | Abcam | ab245235 | 1:500 |
|  |  |  |  |
| Anti-CD21 antibody | Abcam | ab227662 | 1:100 |
|  |  |  |  |
| Anti-Ki67 antibody  Anti-CD4 antibody  Anti-CD8 alpha antibody | Abcam  Abcam  Abcam | ab15580  ab133616  ab237709 | 1:500  1:200  1:100 |
|  |  |  |  |
| Anti-alpha smooth muscle Actin antibody | Abcam | AF801 | 1:100 |
|  |  |  |  |
| PD-1/CD279 Polyclonal antibody | Proteintech | 18106-1-AP | IHC:1:500  IF:1:200 |
|  |  |  |  |
| Cytokeratin 15 Polyclonal antibody | Proteintech | 10137-1-AP | IHC:1:1200  IF:1:200 |
|  |  |  |  |
| p16^INK4a^ Monoclonal Antibody | ZSGB-BIO | ZM-0205 | — |
|  |  |  |  |
| Anti-CD21 antibody [SP186] - C-terminal | Abcam | ab227662 | 1:200 |
|  |  |  |  |
| Anti-CD4 antibody [EPR19514] | Abcam | ab183685 | 1:500 |
|  |  |  |  |
| CD19 Polyclonal antibody | Proteintech | 31263-1-AP | 1:400 |
|  |  |  |  |
| Anti-CD8 alpha antibody [EPR21769] | Abcam | ab217344 | 1:2000 |
|  |  |  |  |
| SOX2 monoclonal antibody | Proteintech | 66411-1-Ig | 1:500 |
|  |  |  |  |
| ZSGB-Bio Mouse/Rabbit Enhanced Polymer Detection System | ZSGB-BIO | PV-9000 | — |
|  |  |  |  |
| Universal DAB Detection Kit | ZSGB-BIO | ZLI-9018 | 1:20 |
|  |  |  |  |
| ZSGB-Bio Rabbit Enhanced Polymer Detection System | ZSGB-BIO | PV-6000 |  |
|  |  |  |  |
| Multi-rAb CoraLite® Plus 488-Goat Anti-Rabbit Recombinant Secondary Antibody (H+L) | Proteintech | RGAR002 | 1:200 |
|  |  |  |  |
| CoraLite594-conjugatedGoat Anti-Mouse IgG(H+L) | Proteintech | SA00013-3 | 1:200 |

**Supplementary Table 2. Signature genes of indicated cell types.**

| Cell Type | Signature Genes |
| --- | --- |
| T cell | CD3D, CD3E |
| Naïve T cell | IL7R, CCR7, CXCR4 |
| GZMK_CD8^+^ T cell | GZMK |
| GZMB_CD8^+^ T cell | GZMB |
| Treg | FOXP3, CTLA4 |
| Tfh | CXCL13 |
| Cycling T cell | MKI67, STMN1 |
| NK | KLRC1, KLRF1, KLRD1 |
| B cell | CD79A, CD19, MS4A1 |
| Plasma cell | CD79A , IGHG1 |
| Myeloid cell | CD79A |
| Macrophage | APOE, CD163, FCGR3A |
| Monocyte | FCN1 |
| Dendritic cell | CD1C, FSCN1 |
| Mast cell | TPSAB1 |
| Plasmacytoid Dendritic Cell | LILRA4 |
| Fibroblast | COL1A2, DCN, FAP |
| Epithelial cell | KRT5, KRT6, KRT13 |
| Endothelial cell | CLDN5, PECAM1, VWF |

**Supplementary Table 3. Clinical information of patient for spatial transcriptome sequencing.**

| Sample | HPV^-^HNSCC-1 | HPV^-^HNSCC-2 | HPV^+^HNSCC-1 | HPV^+^HNSCC-2 |
| --- | --- | --- | --- | --- |
| Disease Diagnosis | Laryngeal Cancer | Laryngeal Cancer | Laryngeal Cancer | Laryngeal Cancer |
| Location | Epiglottis surface | Laryngeal Chamber | Laryngeal Chamber | Aryepiglottic Fold |
| HPV state | Negative | Negative | Positive | Positive |
| Smoke | Yes | Yes | No | Yes |
| Alcohol | Yes | Yes | No | Yes |
| T stage | T3 | T4a | T2 | T2 |
| N stage | N0 | N0 | N0 | N0 |
| M stage | M0 | M1 | M0 | M0 |
| Abbreviations: HPV, human papillomavirus; T, tumor; N, node; M, Metastasis. | | | | |

**Supplementary Table 4.TLS region and TLS-adjacent region in 4 HPV^+^ and HPV^-^HNSCC tissues.**

| Sample | TLS region | TLS-adjacent region |
| --- | --- | --- |
| HPV^+^HNSCC-1 | Cluster10 | Cluster3, Cluster4 |
| HPV^+^HNSCC-2 | Cluster0 | Cluster1, Cluster2 |
| HPV^-^HNSCC-1 | Cluster15 | Cluster 0, Cluster9 |
| HPV^-^HNSCC-2 | Cluster14 | Cluster0 |

**Supplementary Table 5. Pimers for qRT- PCR.**

| Primers name | Sequence (3’-5’) |
| --- | --- |
| KRT15-F | AGACCTGAGACGCACGATG |
| KRT15-R | CGGTAAGTAGCGATCTCCTGC |
| TBP-F | CACGGCACTGATTTTCAGTTCT |
| TBP-R | TTCTTGCTGCCAGTCTGGACT |
| CCL20-F | GGAATGGAATTGGACATAGCC |
| CCL20-R | CCTCCATGATGTGCAAGTGA |
| HPV16 E5-F | TCAGATCTCGAGCTCAAGCTTATGACAAATCTTGATACTGCATCCACAAC |
| HPV16 E5-R | TATCTAGATCCGGTGGATCCTTATGTAATTAAAAAGCGTGCATGTTGTATG |
| HPV16 E6-F | GAACAGCAATACAACAAACCG |
| HPV16 E6-R | CCACCGACCCCTTATATTATG |
| HPV16 E7-F | CAGCTCAGAGGAGGAGGATG |
| HPV16 E7-R | CACAACCGAAGCGTAGAGTC |
