# Supplementary figures and images for: CCL20 secreted by KRT15high tumor Cells promotes tertiary lymphoid structure formation and enhances anti-PD-1 therapy response in HPV+HNSCC
Source: Cell Death Dis. 2025 Dec 29;17(1):150. doi: 10.1038/s41419-025-08359-5 (PMC12858956; doi:10.1038/s41419-025-08359-5)

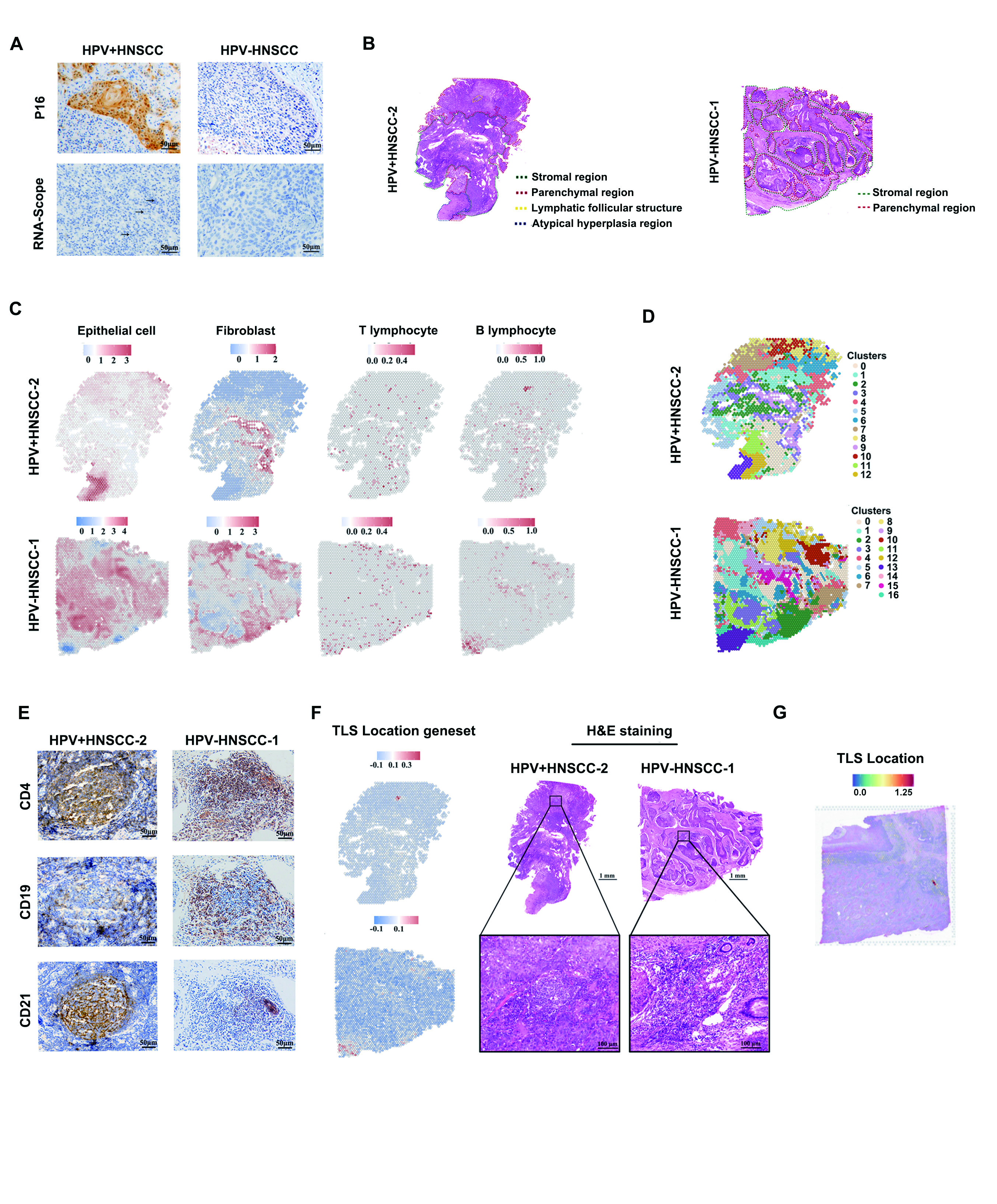

Supplement: Supplementary file 3 — Supplementary Figure 1 [file 41419_2025_8359_MOESM3_ESM.tif]

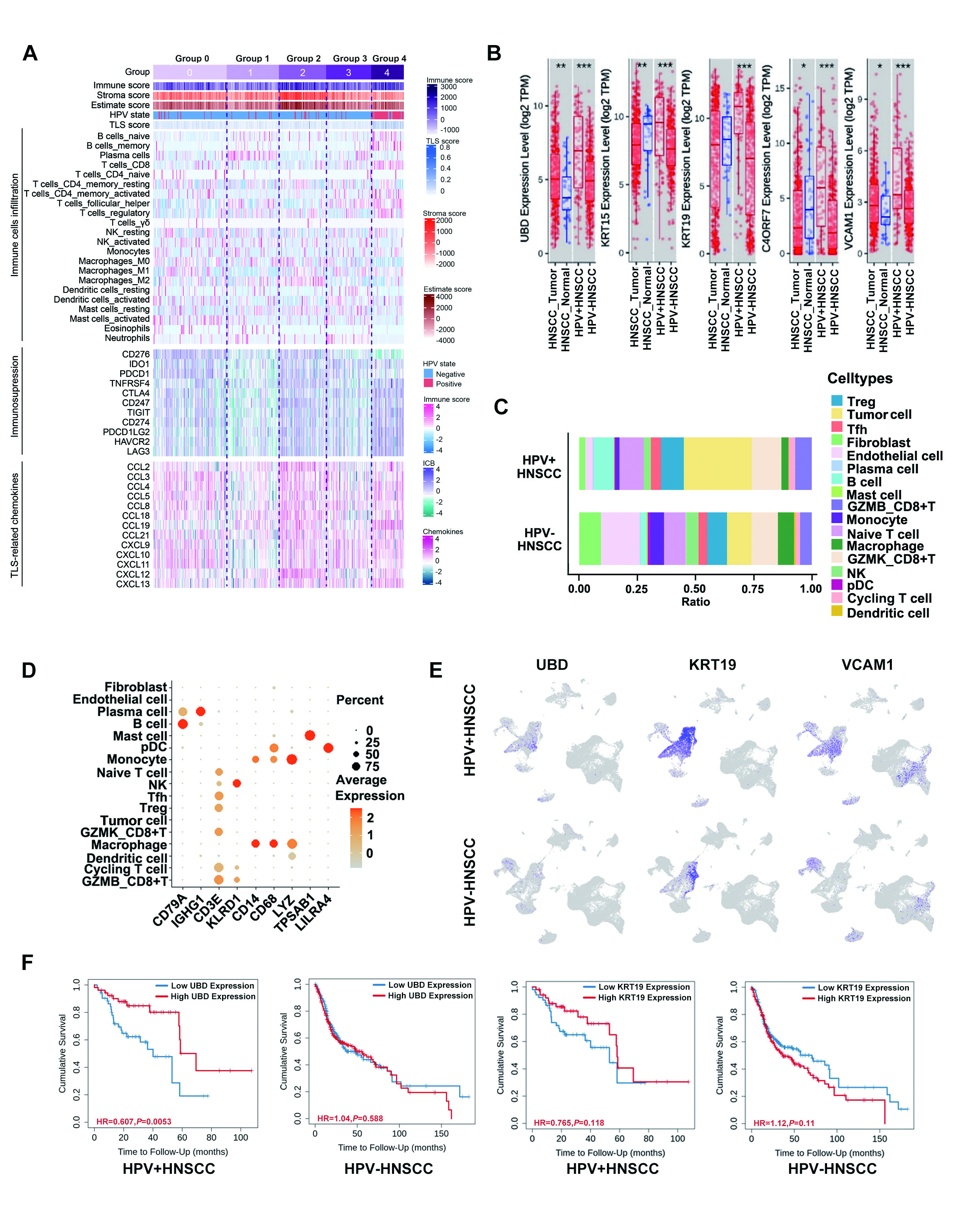

Supplement: Supplementary file 4 — Supplementary Figure 2 [file 41419_2025_8359_MOESM4_ESM.tif]

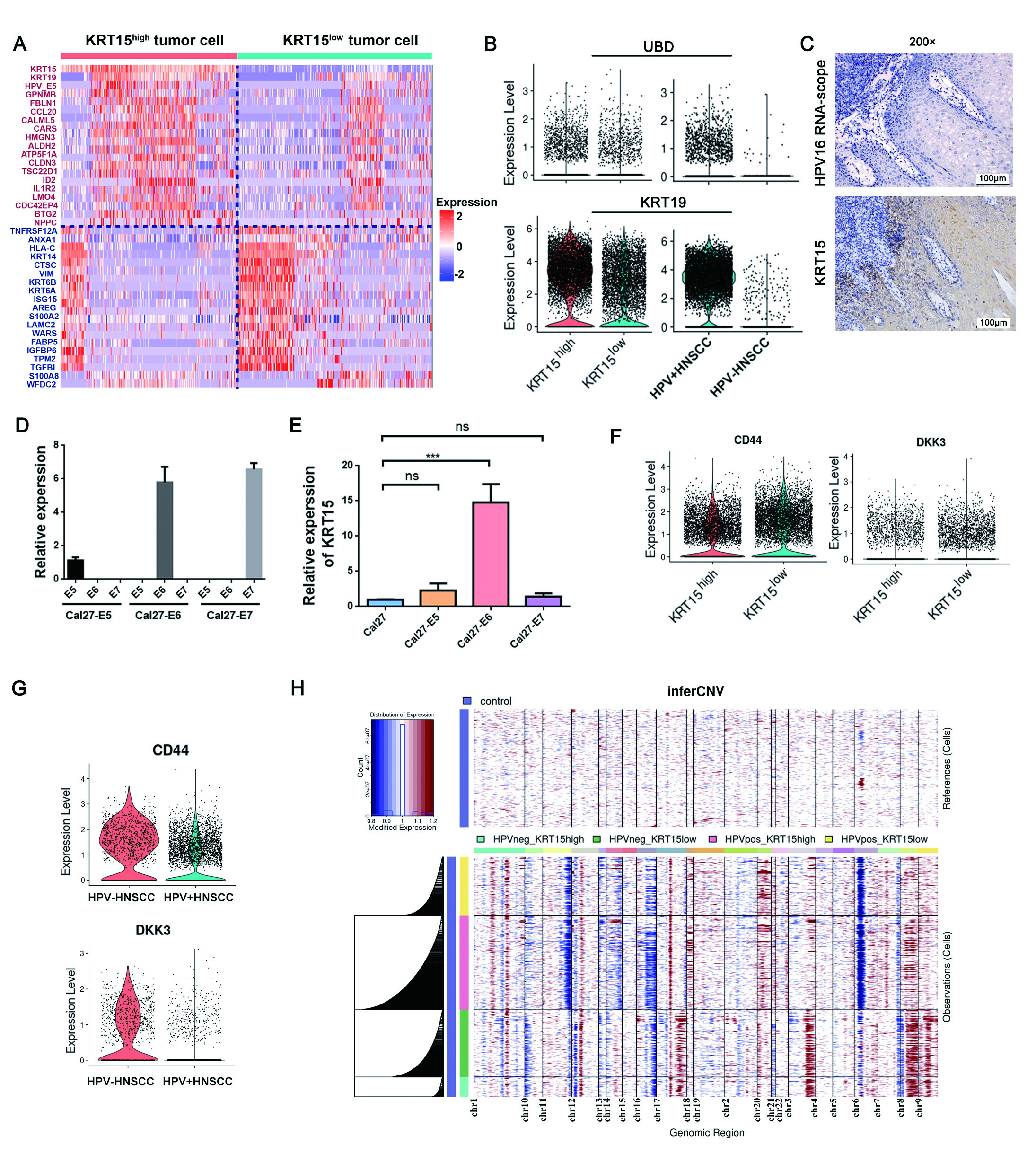

Supplement: Supplementary file 5 — Supplementary Figure 3 [file 41419_2025_8359_MOESM5_ESM.tif]

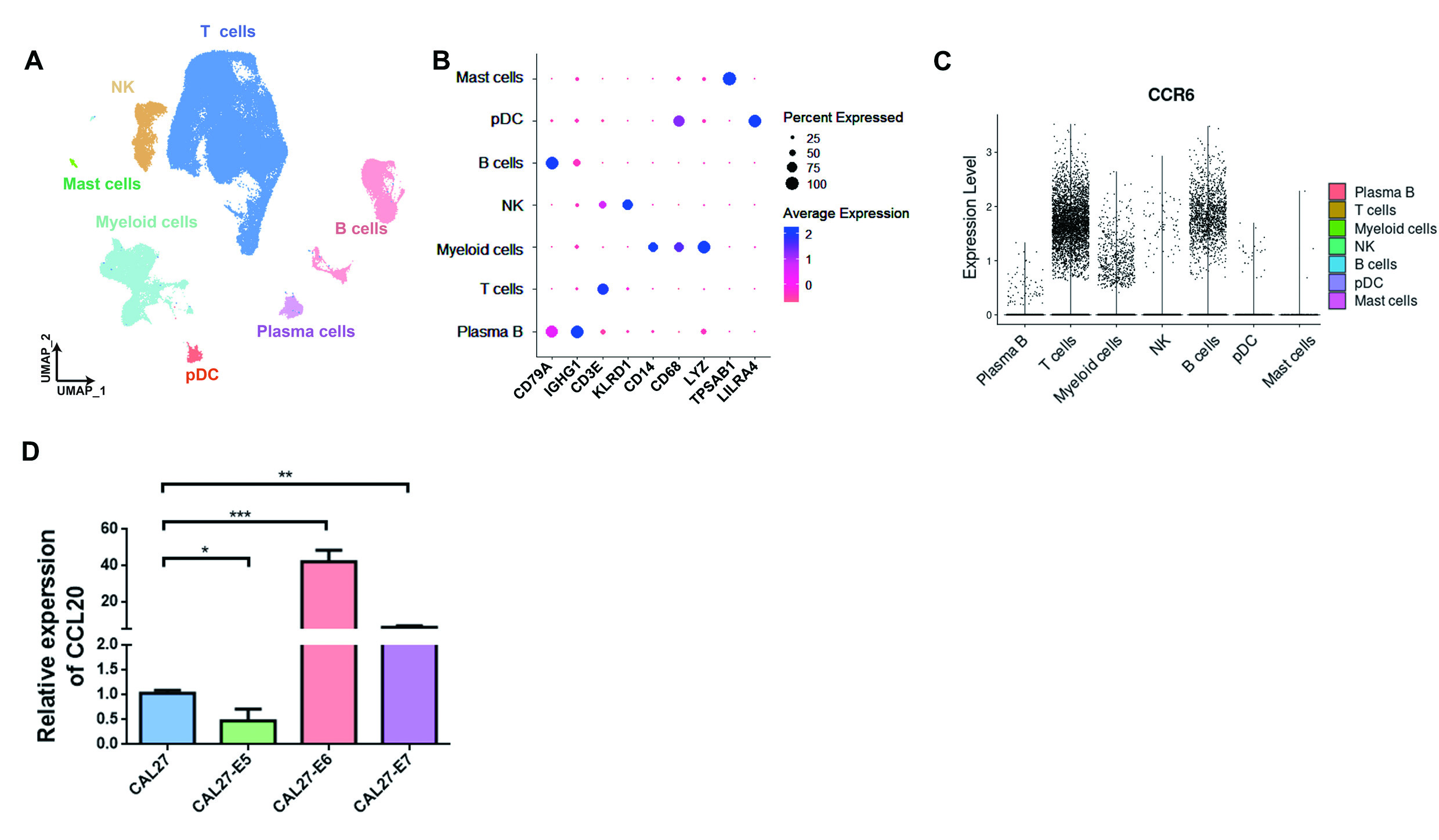

Supplement: Supplementary file 6 — Supplementary Figure 4 [file 41419_2025_8359_MOESM6_ESM.tif]

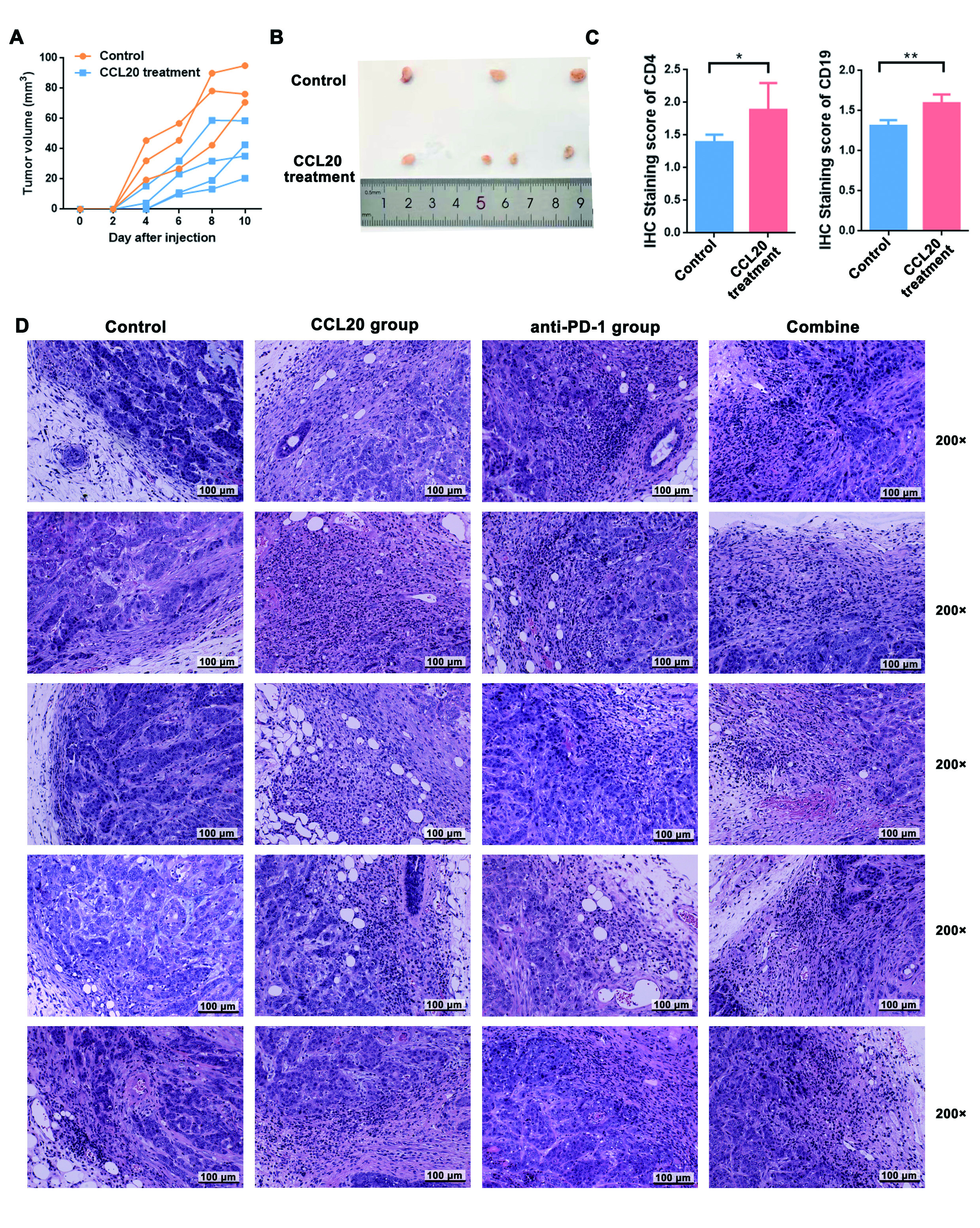

Supplement: Supplementary file 7 — Supplementary Figure 5 [file 41419_2025_8359_MOESM7_ESM.tif]

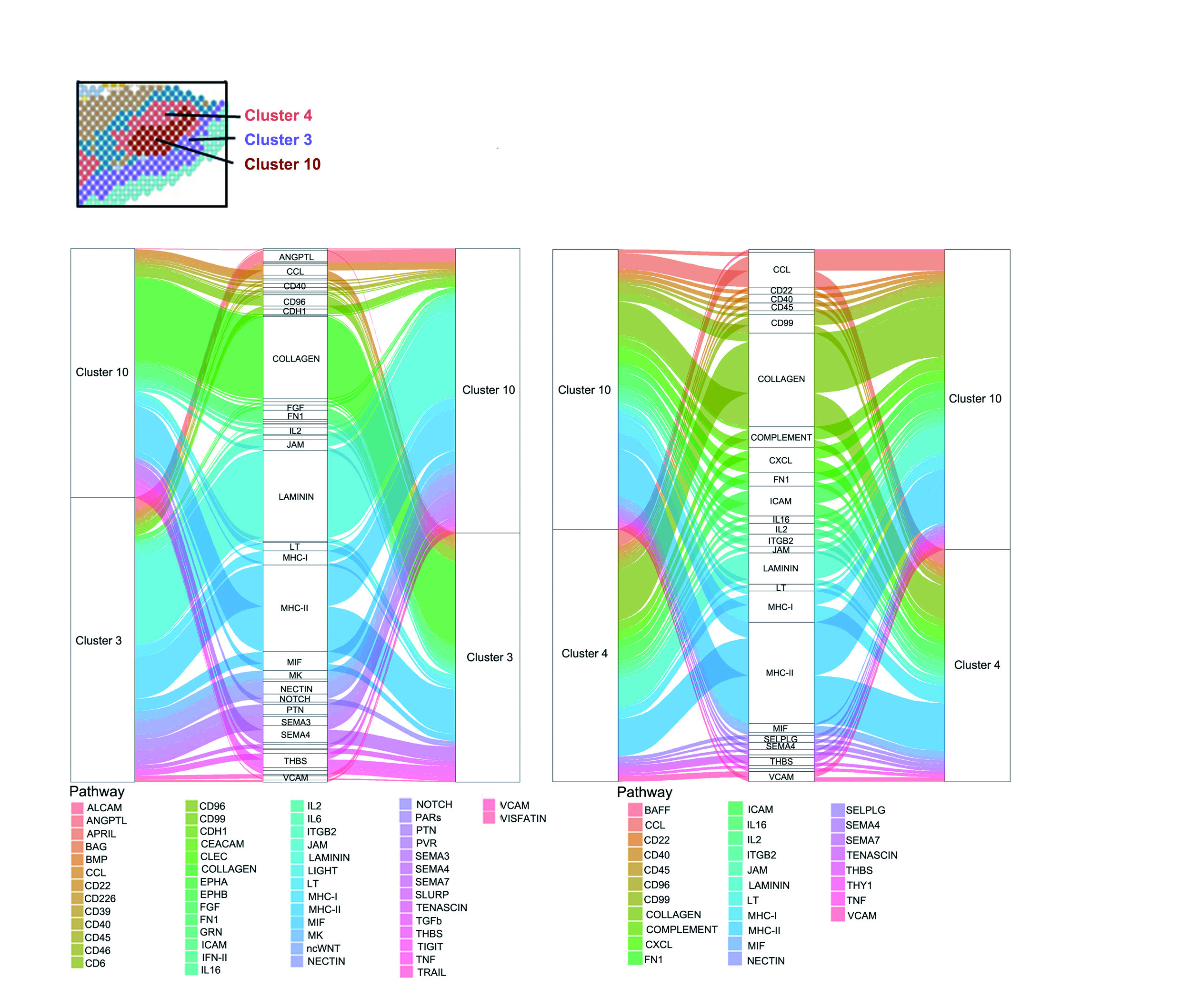

Supplement: Supplementary file 8 — Supplementary Figure 6 [file 41419_2025_8359_MOESM8_ESM.tif]
